# Determining the Association Between Hearing Disability and Injury Risk in Older Adults Using Propensity Score Matching: Quasi-Experimental Study

**DOI:** 10.2196/78826

**Published:** 2026-03-27

**Authors:** Woo-Ri Lee, Sungyoun Chun, Youyoung An, Hyun Seung Choi

**Affiliations:** 1 Division of Cancer Control and Policy, National Cancer Control Institute, National Cancer Center, Goyang, South Korea; 2 Department of Research and Analysis, National Health Insurance Service Ilsan Hospital, Goyang, South Korea; 3 Department of Otorhinolaryngology, National Health Insurance Service Ilsan Hospital, Ilsan-ro 100, Goyang, South Korea, +82 31-900-0114

**Keywords:** hearing disability, injury, fall, older patients, propensity score matching

## Abstract

**Background:**

With rapid population aging, both traumatic injuries and hearing disability have become increasingly prevalent among older adults. Hearing disability may increase vulnerability to injury through impaired balance, reduced environmental awareness, and functional limitations; however, longitudinal evidence examining the association between hearing disability and injury risk remains limited.

**Objective:**

This study aimed to examine the association between hearing disability and the risk of injury among older adults using a quasi-experimental design with propensity score matching (PSM).

**Methods:**

This population-based cohort study included individuals aged 60 years and older with hearing disabilities and a matched control group without disabilities using data from the National Health Insurance Service–Senior cohort from 2008 to 2019. Injury admission, defined using *International Classification of Diseases* codes S00 to S99, was the primary outcome. A quasi-experimental design was applied using PSM at a 1:3 ratio to balance baseline characteristics between the hearing-disabled and nondisabled groups. Cox proportional hazard regression models adjusted for all covariates were used to estimate hazard ratios. Sensitivity analyses were conducted according to disability severity and injury site.

**Results:**

The total number of participants was 43,944, with 10,986 (25%) in the hearing-disabled group and 32,958 (75%) in the nondisabled group, thus confirming a 1:3 matching ratio. The PSM results showed that the standardized mean difference values for all covariates were below the absolute value of 0.1, thus indicating that PSM was successfully performed. The incidence of injury admissions was higher in the hearing-disabled group (1567/10,986, 14.3% of patients) than in the nondisabled group (3966/32,958, 12%), and this difference was statistically significant (*P*<.001). During the follow-up period, older adults with hearing disability had a significantly higher risk of injury admission compared with those without hearing disability (hazard ratio=1.21, 95% CI 1.14‐1.28; *P*<.001). The association was stronger among individuals with more severe hearing disability and varied by injury site.

**Conclusions:**

Hearing disability in older adults is independently associated with increased injury admission risk, with greater severity conferring a higher risk and variation by injury site. Interventions such as hearing aid provision, targeted traffic safety measures, and enhanced community and family support are warranted to mitigate this burden.

## Introduction

The proportion of older individuals is increasing worldwide. By 2050, individuals aged 65 years and older are projected to account for approximately 15.9% of the total population [1]. In South Korea, the proportion of older adults has exceeded 20% as of 2024, thus officially classifying the country as a superaged society [2]. As a consequence of this demographic shift, traumatic injury in older adults has emerged as a significant public health concern [3]. Geriatric injuries are characterized by distinct epidemiological patterns in which ground-level falls and motor vehicle crashes are the predominant mechanisms of injury, frequently resulting in predictable injury types such as fractures (particularly of the hip and spine), head trauma, and thoracic injuries among older adults [4]. A study that analyzed the trends in trauma incidence using the National Trauma Data Bank (United States) and TraumaRegister DGU (Germany) reported an increasing trend in geriatric injury cases in both datasets [5]. According to data from the Korea Centers for Disease Control and Prevention, approximately 320,000 older adults sustained injuries in 2024, which accounted for 38.4% of all injury cases in the country [6]. Prior studies have identified traffic accidents and falls as the leading causes of injury among older adults [3,7-9], with geriatric injury being one of the major contributors to preventable mortality [10].

In parallel with the aging population, the prevalence of hearing disability is also increasing worldwide [11]. In South Korea, approximately 24.9% of registered individuals with disabilities aged 65 years and older had a hearing disability as of 2023, and this number is expected to continue growing [12]. Hearing disability in older adults extends beyond auditory difficulties and negatively affects physical and social functioning [11]. From a functional perspective, hearing impairment may influence balance control and situational awareness, which are essential for injury prevention in daily life. One study demonstrated that individuals with hearing loss experience reduced awareness of environmental sounds and warning signals while walking, thereby increasing the risk of falls [12]. Additionally, the social and psychological consequences of hearing loss, including isolation and depression, are associated with decreased physical activity, which may further impair balance and gait function [13]. Current studies have reported that consistent hearing aid use is associated with a lower prevalence and risk of falls among older adults, suggesting a potential link between hearing-related functional limitations and injury risk [14]. Furthermore, longitudinal evidence indicates that hearing loss is associated with an increased risk of falls and fall-related injuries in older populations [15].

However, the number of studies directly examining the association between hearing disability and injury in older adults remains limited. Most studies have used cross-sectional designs or relatively small sample sizes, thus restricting the ability to establish causal inferences [16-18]. Furthermore, many studies have had limited ability to adequately control for baseline differences between individuals with and without hearing disability. Therefore, this study aimed to examine the association between hearing disability and the occurrence of injury among older adults, and we hypothesized that older adults with hearing disability would have a higher risk of injury than those without hearing disability.

## Methods

### Study Data

We used the National Health Insurance Service (NHIS)–Senior cohort data provided by the NHIS between 2002 and 2019. South Korea operates a single-payer national health insurance (NHI) system; the NHIS, as the sole insurer, provides NHI coverage for 97% of the population and Medical Aid program coverage for the remaining 3%, thus ensuring universal health care coverage [19]. As NHIS enrollees, all citizens pay monthly insurance premiums based on their income and assets. When receiving medical services, patients pay a portion of their total medical expenses as out-of-pocket payments, and the remaining costs are covered by the NHIS as copayments to medical institutions. To receive copayments, medical institutions must submit reimbursement claims to the NHIS. Given that all medical institutions in South Korea must submit claims to the NHIS, the NHIS database comprehensively stores and manages all medical service use records [20,21]. Therefore, NHIS data can be considered nationally representative. The NHIS-Senior cohort was established using a stratified random sampling method. In 2008, the older adult population aged 60 to 80 years was stratified by age, sex, health insurance premium, and region, and 8% of the individuals were randomly selected from each stratum [22]. Accordingly, in this study, older adults were defined as individuals aged 60 years or older in accordance with the sampling framework of the NHIS-Senior cohort.

### Variables

#### Outcome Measures

The main outcome measure of this study was injury admission. Injury admissions were recorded between 2008 and 2019. Injury admission was defined based on the 10th revision of the *International Classification of Diseases*, where hospitalization with principal diagnosis codes S00 to S99 was classified as an injury admission.

#### Independent Variable

The key independent variable in this study was hearing disability status. A diagnosis of hearing disability is defined in the Medical Service Act in South Korea and requires an evaluation by an otolaryngologist at a medical institution equipped with a soundproof booth [23]. Hearing disability is classified based on pure-tone audiometry thresholds at 4 frequencies (0.5, 1, 2, and 4 kHz); hearing loss of 80 dB or higher in one ear and 40 dB or higher in the other ear qualifies as hearing disability, and thresholds of 80 dB or higher in both ears indicate severe hearing disability [23]. The severity of disability in the Korea National Disability Registration System (KNDRS) is classified into 6 grades; for hearing disability, grades 2 and 3 indicate severe impairment, and grades 4 to 6 indicate mild impairment [23,24].

Since 1989, South Korea has used the KNDRS to register individuals with disabilities [23]. The NHIS database has been linked to the KNDRS to provide disability status information. Therefore, in this study, hearing disability status was identified using linked data [23].

#### Covariates

The following variables were used as covariates: sex (male and female), age group (60s, 70s, and >80 years), income level (quintile), type of health care insurance (Medical Aid, NHI self-employed, and NHI employee), region (Seoul, Gyeonggi, metropolitan, and rural), Charlson Comorbidity Index score (0, 1, 2, and ≥3), and index year. Age was included as a categorical variable to improve interpretability, account for potential nonlinear associations, and facilitate covariate balance in the propensity score matching (PSM).

### Statistical Analysis

The analysis was conducted in 5 steps. First, PSM was performed to ensure homogeneity between the case and control groups. PSM is a quasi-experimental method that reduces selection bias and mimics a randomized clinical trial in observational studies [25]. By using binomial logistic regression, PSM calculates the probability value corresponding to the independent variable. The computed probability value was referred to as the propensity score. Matching was performed using the greedy method at a 1:3 ratio with replacement, with a caliper of 0.1. Exact matching was not applied. All the covariates included in the study (sex, age, income, type of health care insurance, region, Charlson Comorbidity Index, and index year) were matched. After PSM, standardized mean difference values below an absolute value of 0.1 were considered to indicate homogeneity between the case and control groups. For binary variables, standardized mean difference values were calculated as the standardized differences in proportions between groups. Second, the chi-square test was used to examine the relationship between the participants’ general characteristics and injury admission. Statistical significance was set at a value of *P* below .05. Third, survival periods and incidence rates were calculated according to the participants’ general characteristics. Differences in survival periods were assessed using 2-tailed *t* tests and ANOVA, and statistical significance was set at a value of *P*<.05. The incidence rates were calculated as the number of events per 100,000 person-days. Fourth, regression analysis using the Cox proportional hazard model was conducted to evaluate the association between hearing disability and injury admission. The proportional hazard assumption was verified using Kaplan-Meier survival curves, and the differences in survival curves according to hearing disability status were tested using the log-rank test. Statistical significance for the log-rank test and Cox regression analysis was set at a value of *P*<.05. Finally, sensitivity analysis was performed to enhance the robustness of the results. Sensitivity analysis was conducted using two approaches: (1) subgroup analysis, in which hearing disability was classified into mild and severe and wherein each subgroup was compared with the control group; and (2) site-specific injury analysis, in which injury admissions were categorized based on the affected body region. All regression analyses were adjusted for all covariates used in PSM to improve robustness [25]. All analyses were performed using SAS (version 9.4; SAS Institute) and R software (version 3.6.3; R Foundation for Statistical Computing).

### Ethical Considerations

Ethical approval for this study was waived by the institutional review board of the NHIS Ilsan Hospital, South Korea (NHIMC 2025-03-027), because the study used secondary data containing anonymized and encrypted personal information. The requirement for informed consent was also waived. The data were analyzed in a deidentified form to protect the privacy and confidentiality of individuals. As the study used anonymized secondary data, informed consent and participant compensation were not applicable.

## Results

**Figure 1.**
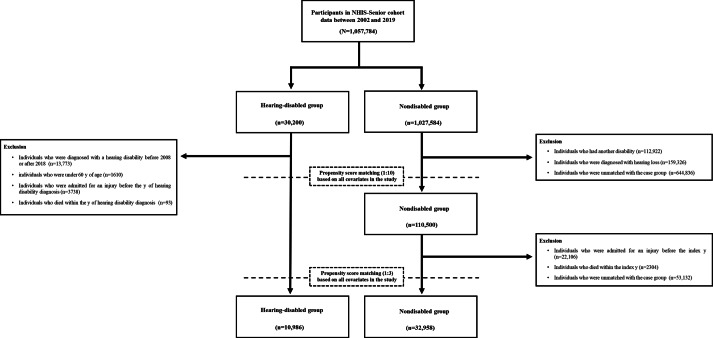
Participant selection flowchart. NHIS: National Health Insurance Service.

The NHIS-Senior cohort initially included 1,057,784 individuals. The hearing-disabled group (case group) and nondisabled group (control group) consisted of 30,200 and 1,027,584 individuals, respectively. The exclusion criteria were applied to define the final study population. In the nondisabled group, the following individuals were excluded: those with other disabilities (112,922/1,027,584, 11%), those with a history of hearing loss (159,326/1,027,584, 15.5%), those excluded from PSM for the index year matching (644,836/1,027,584, 62.8%), those with injury-related hospital admissions before the index year (22,106/1,027,584, 2.2%), those who died in the index year (2304/1,027,584, 0.2%), and those further excluded from PSM (53,132/1,027,584, 5.2%). In the hearing-disabled group, the following individuals were excluded: those diagnosed with hearing disability before 2008 or after 2018 (13,773/30,200, 45.6%), those younger than 60 years (1610/30,200, 5.3%), those with injury-related hospital admissions before hearing disability diagnosis (3738/30,200, 12.4%), and those who died within the year of hearing disability diagnosis (93/30,200, 0.3%). After applying the exclusion criteria, the final study population included 10,986 and 38,411 patients in the hearing-disabled and nondisabled group, respectively (Figure 1).

Multimedia Appendix 1 shows the general characteristics of the study population before and after PSM. After PSM, the total number of study participants was 43,944, with 10,986 (25%) in the hearing-disabled group and 32,958 (75%) in the nondisabled group, thus confirming a 1:3 matching ratio. The PSM results showed that the standardized mean difference values for all covariates were below the absolute value of 0.1, thus indicating that PSM was successfully performed.

Table 1 shows the relationships between the participants’ general characteristics and admissions for injury. Of the 43,944 participants, 5533 (12.6%) experienced admissions for injuries. The incidence of injury admissions was higher in the hearing-disabled group (1567/10,986, 14.3% of the participants) than in the nondisabled group (3966/32,958, 12%), and this difference was statistically significant (*P*<.001).

**Table 1.** General characteristics of the study population.

Variables	Injury admission, n (%)		*P* value^a^
	Yes	No	
Total (n=43,944)	5533 (12.6)	38,411 (87.4)	—^b^
Hearing disability			<.001
Nondisabled (n=32,958)	3966 (12.0)	28,992 (88.0)	
Disabled (n=10,986)	1567 (14.3)	9419 (85.7)	
Severity of hearing disability			<.001
Nondisabled (n=32,750)	3932 (12.0)	28,818 (88.0)	
Mildly disabled (n=9923)	1340 (13.5)	8583 (86.5)	
Severely disabled (n=1271)	261 (20.5)	1010 (79.5)	
Sex			<.001
Male (n=24,348)	2354 (9.7)	21,994 (90.3)	
Female (n=19,596)	3179 (16.2)	16,417 (83.8)	
Age (years)			<.001
60s (n=11,355)	1363 (12.0)	9992 (88.0)	
70s (n=20,660)	2865 (13.9)	17,795 (86.1)	
Older than 80 (n=11,929)	1305 (10.9)	10,624 (89.1)	
Income			.20
Quintile 1 (lowest; n=10,565)	1397 (13.2)	9168 (86.8)	
Quintile 2 (n=4326)	531 (12.3)	3795 (87.7)	
Quintile 3 (n=5689)	713 (12.5)	4976 (87.5)	
Quintile 4 (n=8242)	1042 (12.6)	7200 (87.4)	
Quintile 5 (highest; 15,122)	1850 (12.2)	13,272 (87.8)	
Type of health care insurance			<.001
Medical Aid (n=3976)	601 (15.1)	3375 (84.9)	
NHI^c^ self-employed (n=11,525)	1496 (13.0)	10,029 (87.0)	
NHI employee (n=28,443)	3436 (12.1)	25,007 (87.9)	
Region			<.001
Seoul (n=6837)	748 (10.9)	6089 (89.1)	
Gyeonggi (n=7296)	783 (10.7)	6513 (89.3)	
Metropolitan (n=11,238)	1185 (10.5)	10,053 (89.5)	
Rural (n=18,573)	2817 (15.2)	15,756 (84.8)	
CCI^d^			<.001
0 (n=17,663)	2234 (12.6)	15,429 (87.4)	
1 (n=9511)	1298 (13.6)	8213 (86.4)	
2 (n=6802)	804 (11.8)	5998 (88.2)	
≥3 (n=9968)	1197 (12.0)	8771 (88.0)	
Index year			<.001
2008 (n=4753)	1181 (24.8)	3572 (75.2)	
2009 (n=4899)	1143 (23.3)	3756 (76.7)	
2010 (n=4066)	829 (20.4)	3237 (79.6)	
2011 (n=1814)	323 (17.8)	1491 (82.2)	
2012 (n=1246)	212 (17.0)	1034 (83.0)	
2013 (n=1248)	168 (13.5)	1080 (86.5)	
2014 (n=1227)	165 (13.4)	1062 (86.6)	
2015 (n=1564)	175 (11.2)	1389 (88.8)	
2016 (n=5699)	542 (9.5)	5157 (90.5)	
2017 (n=7477)	456 (6.1)	7021 (93.9)	
2018 (n=9951)	339 (3.4)	9612 (96.6)	

a *P *values were obtained using the chi-square test.

b Not applicable.

c NHI: national health insurance.

d CCI: Charlson Comorbidity Index.

Table 2 shows the analysis comparing injury admission incidence rates and survival times according to the participants’ general characteristics. The overall incidence rate was 8.4 cases per 100,000 person-days, and the mean survival time was 1497 (SD 1230) days. The hearing-disabled group had an incidence rate of 9.7 cases per 100,000 person-days, which was higher than that in the nondisabled group (8.0 cases per 100,000 person-days). However, the difference in survival time between the 2 groups was not statistically significant.

**Table 2.** Incidence rate per 100,000 person-years and survival time.

Variables	Injury admission		*P* value
	Incidence rate	Survival time (days), mean (SD)	
Total	8.4	1497 (1230)	—^a^
Hearing disability			.051
Nondisabled	8.0	1503 (1236)	
Disabled	9.7	1477 (1212)	
Hearing disability severity			< .001
Nondisabled	8.0	1505 (1237)	
Mildly disabled	9.5	1423 (1190)	
Severely disabled	11.0	1864 (1292)	
Sex			.08
Male	6.4	1506 (1226)	
Female	10.9	1485 (1235)	
Age (y)			< .001
60s	5.8	2081 (1336)	
70s	9.0	1545 (1223)	
Older than 80	12.8	856 (737)	
Income			< .001
Quintile 1 (lowest)	9.0	1474 (1211)	
Quintile 2	8.0	1529 (1230)	
Quintile 3	8.1	1555 (1255)	
Quintile 4	8.0	1582 (1270)	
Quintile 5 (highest)	8.5	1435 (1208)	
Type of health care insurance			< .001
Medical Aid	11.0	1371 (1173)	
NHI^b^ self-employed	8.3	1571 (1254)	
NHI employee	8.1	1484 (1226)	
Region			< .001
Seoul	6.8	1620 (1275)	
Gyeonggi	7.2	1499 (1240)	
Metropolitan	7.4	1426 (1222)	
Rural	10.2	1493 (1211)	
CCI^c^			< .001
0	7.3	1725 (1301)	
1	8.9	1527 (1218)	
2	8.7	1364 (1173)	
≥3	10.4	1153 (1046)	
Index year			< .001
2008	8.3	2982 (1300)	
2009	8.5	2752 (1176)	
2010	7.9	2572 (1020)	
2011	7.7	2327 (902)	
2012	8.2	2082 (765)	
2013	7.2	1876 (590)	
2014	8.4	1605 (459)	
2015	8.4	1325 (328)	
2016	9.5	1002 (235)	
2017	8.8	692 (125)	
2018	9.5	359 (39)	

a Not applicable.

b NHI: national health insurance.

c CCI: Charlson Comorbidity Index.

**Figure 2.**
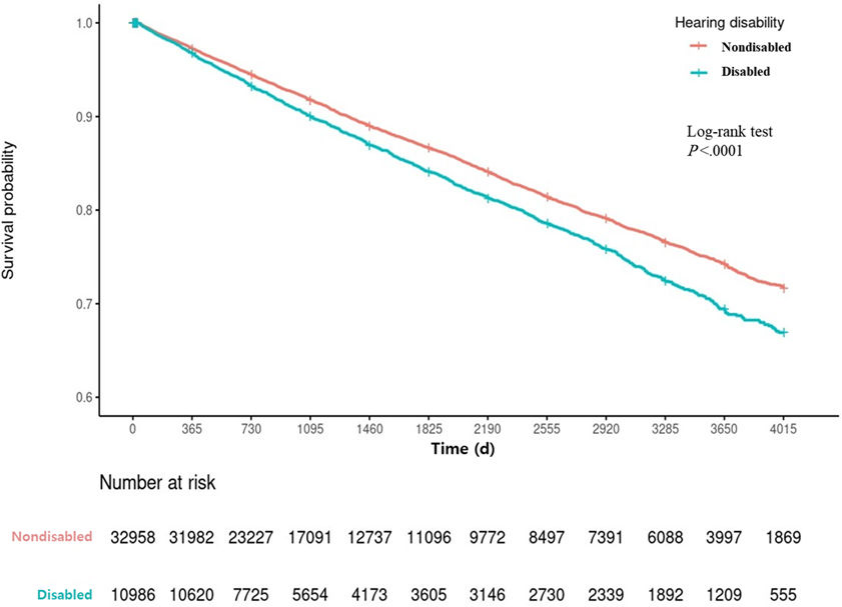
Kaplan-Meier survival curve according to hearing disability status.

Table 3 shows the Cox regression analysis exploring the association between hearing disability and injury admission. The Kaplan-Meier survival curve demonstrated a statistically significant difference in the according to hearing disability status (Figure 2; *P*<.001). The Cox regression analysis indicated that the injury admission risk was higher in the hearing-disabled group than in the nondisabled group (hazard ratio [HR] 1.21, 95% CI 1.14‐1.28).

**Table 3.** Results of the multivariable Cox regression analysis on the association between hearing disability and injury admission.

Variables	Injury admission, HR^a^ (95% CI)	*P* value
Hearing disability		
Nondisabled	1.00 (reference)	—^b^
Disabled	1.21 (1.14‐1.28)	<.001
Sex		
Male	1.00 (reference)	—
Female	1.56 (1.48‐1.65)	< .001
Age (years)		
60s	1.00 (reference)	—
70s	1.49 (1.39‐1.59)	< .001
Older than 80	2.11 (1.93‐2.29)	< .001
Income		
Quintile 1 (lowest)	1.00 (reference)	—
Quintile 2	1.05 (0.94‐1.17)	.40
Quintile 3	1.03 (0.93‐1.14)	.57
Quintile 4	1.04 (0.95‐1.14)	.37
Quintile 5 (highest)	1.02 (0.94‐1.11)	.70
Type of health care insurance		
Medical Aid	1.00 (reference)	—
NHI^c^ self-employed	0.89 (0.79‐0.99)	.04
NHI employee	0.87 (0.78‐0.97)	.01
Region		
Seoul	1.00 (reference)	—
Gyeonggi	1.05 (0.95‐1.17)	.30
Metropolitan	1.09 (0.99‐1.20)	.06
Rural	1.42 (1.31‐1.54)	< .001
CCI^d^		
0	1.00 (reference)	—
1	1.19 (1.11‐1.28)	< .001
2	1.18 (1.09‐1.28)	< .001
≥3	1.40 (1.30‐1.50)	< .001
Index year	1.00 (0.99‐1.01)	.79

a HR: hazard ratio.

b Not applicable.

c NHI: national health insurance.

d CCI: Charlson Comorbidity Index.

**Figure 3.**
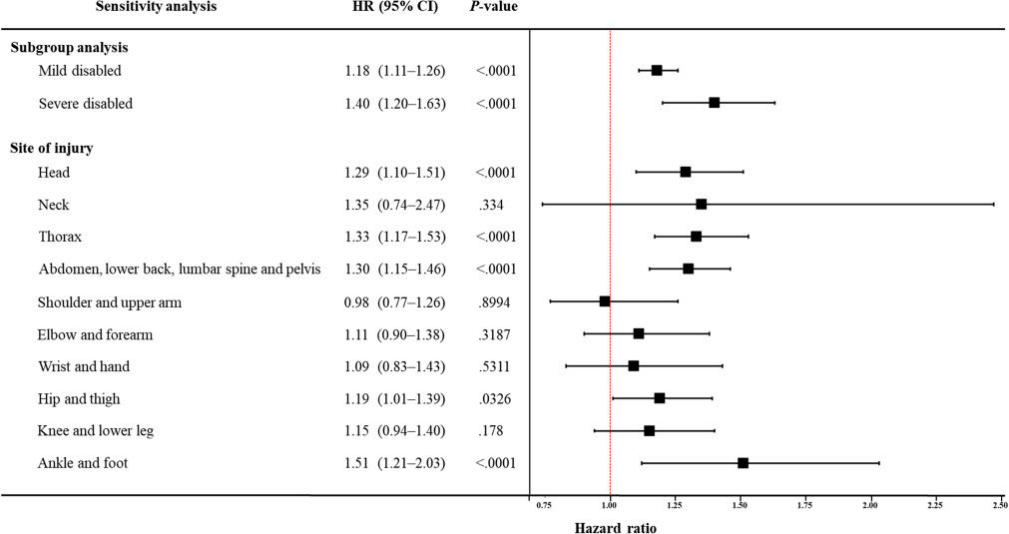
Forest plot of sensitivity analysis results. HR: hazard ratio.

A sensitivity analysis was conducted to enhance the robustness of the findings (Figure 3). The results showed that individuals with hearing disability had a higher injury admission risk regardless of severity (mild disability: HR 1.18, 95% CI 1.11‐1.26; severe disability: HR 1.40, 95% CI 1.20‐1.63). A further analysis of site-specific injury risk revealed statistically significant differences across multiple body regions (head: HR 1.29, 95% CI 1.10‐1.51; thorax: HR 1.33, 95% CI 1.17‐1.53; abdomen, lower back, lumbar spine, and pelvis: HR 1.30, 95% CI 1.15‐1.46; hips and thighs: HR 1.19, 95% CI 1.01‐1.39; ankles and feet: HR 1.51, 95% CI 1.21‐2.03). These anatomical injury sites commonly correspond to clinically meaningful injury types in geriatric populations, such as head injuries, fractures of the hip or lower extremities, and other fall-related blunt injuries. Such injury patterns are consistent with mechanisms frequently observed among older adults, particularly falls and traffic-related trauma.

## Discussion

### Principal Findings

This study aimed to explore the association between hearing disability and injury admissions among older adults. The analysis revealed that older adults with hearing disability were at a significantly higher risk of injury. Moreover, the association was more pronounced with greater disability severity, and the influence varied according to the injury site.

### Interpretation

#### Mechanisms Linking Hearing Disability and Injury

Several mechanisms may explain the observed association between hearing disability and injury risk among older adults. Hearing disability has been associated with accelerated cognitive decline, including impairments in memory, attention, and executive function [26,27]. Such cognitive changes may reduce the ability to detect environmental hazards and appropriately respond to warning signals, thereby increasing susceptibility to falls and other injuries [12]. In addition, auditory deprivation may compromise balance control and spatial orientation, which are essential for safe mobility during daily activities, further increasing injury risk [13]. Taken together, these mechanisms suggest that addressing cognitive and balance-related impairments associated with hearing disability, as well as improving hearing levels, may represent an important component of injury prevention strategies for older adults.

#### Hearing Aid Use and Barriers to Adoption

Functional restoration of auditory capacity may help reduce injury risk among older adults with hearing disability. Hearing aid use has been shown to improve auditory perception as well as depressive symptoms, emotional stability, and social functioning [28-30]. A previous study further indicates that hearing aid use can improve social relationships and overall quality of life among older adults with age-related hearing loss, highlighting social engagement as an important pathway through which auditory rehabilitation may influence broader health outcomes [31]. By enhancing environmental awareness and reducing cognitive load, hearing aids may also decrease the risk of falls and related injuries [32]. Despite these benefits, approximately 30% of individuals with hearing disability in South Korea do not use hearing aids [33]. Barriers to hearing aid use may include financial burden, stigma associated with device use, discomfort, and limited awareness of potential benefits [31,34]. Addressing these barriers may be critical for reducing injury risk in this population. These findings suggest that promoting hearing aid adoption may represent a practical intervention to help reduce injury risk in this vulnerable population.

#### Psychosocial Pathways and Social Support

Beyond direct sensory and functional impairments, hearing disability in older adults is also associated with psychosocial challenges, including social isolation and strained interpersonal relationships [28,35-38]. Reduced social engagement may lead to lower levels of physical activity and decreased participation in daily routines, both of which are known risk factors for falls and injury. Previous studies have shown that stronger social relationships within families and communities are associated with lower rates of fall-related injuries among older adults [39,40]. These findings suggest that social support may play a protective role in mitigating injury risk among individuals with hearing disability. Accordingly, interventions that strengthen social support and promote social engagement may help reduce injury risk in older adults with hearing disability.

#### Injury Patterns and Traffic-Related Injuries

As the older adult population continues to grow, the incidence of traffic-related injuries among this population is also expected to increase [41]. Older adults are particularly vulnerable to traffic-related injuries due to slower reaction times, sensory impairments, and a higher likelihood of being pedestrians. Previous studies have identified common injury sites in traffic accidents, including the head, thorax, abdomen, pelvis, and lower extremities [42,43]. Consistent with these findings, our study observed elevated injury risks in older adults with hearing impairment across multiple body regions, including the head, trunk, pelvis, and lower extremities. Furthermore, pedestrians with sensory disabilities, including hearing loss, have been shown to have a higher risk of traffic-related injury and mortality [44]. Reduced perception of environmental sounds and warning signals may further increase vulnerability to traffic accidents among individuals with hearing disability. Together, these findings underscore the importance of incorporating sensory impairment considerations into traffic safety policies and pedestrian protection strategies for older adults.

#### Implications for Health Care and Policy

These findings have important implications for health care systems and public health policy. First, routine hearing screening and early identification of hearing disability in older adults may facilitate the timely recognition of individuals at increased risk of injury. Second, integrating hearing assessment into fall prevention programs and geriatric care pathways could enable more comprehensive risk management and contribute to reductions in injury-related morbidity by addressing both sensory and functional vulnerabilities. Third, improving access to hearing aids and promoting their use through financial support and public awareness campaigns may serve as practical and effective strategies to mitigate injury risk at the individual level. Finally, beyond clinical and community-based interventions, system-level approaches are also warranted. Targeted traffic safety interventions—such as pedestrian-friendly road design and enhanced auditory warning systems—may further help protect older adults with hearing disability and reduce traffic-related injuries.

### Limitations

This study has a few limitations. First, owing to the nature of the claims data, only primary disabilities could be identified, and information on multiple co-occurring disabilities was unavailable. Consequently, individuals with other severe types of disability and mild hearing impairment could not be classified as hearing disabled and were excluded from the analysis. Future studies should incorporate methods that detect and include participants with multiple disabilities. Second, we could not directly ascertain whether the injuries resulted from traffic accidents. Given that injury admissions were defined solely by *International Classification of Diseases, 10th Revision*, diagnosis codes, we were unable to distinguish traffic-related trauma from other causes. To address this limitation, we conducted subgroup analyses according to injury site and compared our results with the major injury locations reported in traffic accident studies. Third, some older adults may have experienced improvements in auditory function through hearing aid use during follow-up; however, based on the available data, we could not assess hearing aid adoption or auditory improvement. Despite these limitations, this study is meaningful because it confirmed the association between hearing disability status and injury incidence among older adults using a longitudinal design.

### Conclusions

Our findings demonstrated a clear disparity in injury risk according to hearing disability status in older adults. To reduce these health disparities, it is essential to promote hearing aid use, implement targeted traffic safety measures, and strengthen social support at both the community and family levels.

## Supplementary material

10.2196/78826 Multimedia Appendix 1 General characteristics of the study population before and after propensity score matching.

## Abbreviations

HR: hazard ratio
KNDRS: Korea National Disability Registration System
NHI: national health insurance
NHIS: National Health Insurance Service
PSM: propensity score matching
