# Supplementary material for: Determining the Association Between Hearing Disability and Injury Risk in Older Adults Using Propensity Score Matching: Quasi-Experimental Study
Source: JMIR Public Health Surveill. 2026 Mar 27;12:e78826. doi: 10.2196/78826 (PMC13026427; doi:10.2196/78826)
Supplement: Multimedia Appendix 1 [file publichealth-v12-e78826-s001.docx]

| **Table S1. General characteristics of the study population before and after propensity score matching** | | | | | | | | | |  |
| --- | --- | --- | --- | --- | --- | --- | --- | --- | --- | --- |
| **Variable** | **Before PSM** | | | | **After PSM** | | | | | |
|  | **Non-disabled** | | **Disabled** | | **Non-disabled** | | **Disabled** | | **Standardized  mean  difference** | |
|  | **N** | **%** | **No** | **%** | **N** | **%** | **No** | **%** |  |  |
| **Total** | 86,090 | (88.7) | 10,986 | (11.3) | 32,958 | (75.0) | 10,986 | (25.0) |  | |
| **Sex** |  |  |  |  |  |  |  |  |  | |
| Male | 49,707 | (89.1) | 6,088 | (10.9) | 18,260 | (75.0) | 6,088 | (25.0) |  | |
| Female | 36,383 | (88.1) | 4,898 | (11.9) | 14,698 | (75.0) | 4,898 | (25.0) | 0.00024 | |
| **Age** |  |  |  |  |  |  |  |  |  | |
| 60s | 24,302 | (89.5) | 2,839 | (10.5) | 8,516 | (75.0) | 2,839 | (25.0) |  | |
| 70s | 41,555 | (88.9) | 5,163 | (11.1) | 15,497 | (75.0) | 5,163 | (25.0) | 0.00049 | |
| Over 80s | 20,233 | (87.1) | 2,984 | (12.9) | 8,945 | (75.0) | 2,984 | (25.0) | -0.00049 | |
| **Income** |  |  |  |  |  |  |  |  |  | |
| Q1 (lowest) | 20,487 | (88.6) | 2,639 | (11.4) | 7,926 | (75.0) | 2,639 | (25.0) |  | |
| Q2 | 8,468 | (88.7) | 1,082 | (11.3) | 3,244 | (75.0) | 1,082 | (25.0) | -0.00020 | |
| Q3 | 11,150 | (88.7) | 1,423 | (11.3) | 4,266 | (75.0) | 1,423 | (25.0) | -0.00027 | |
| Q4 | 16,287 | (88.8) | 2,061 | (11.2) | 6,181 | (75.0) | 2,061 | (25.0) | -0.00016 | |
| Q5 (highest) | 29,698 | (88.7) | 3,781 | (11.3) | 11,341 | (75.0) | 3,781 | (25.0) | -0.00013 | |
| **Type of healthcare insurance** |  |  |  |  |  |  |  |  |  | |
| Medical Aid | 7,446 | (88.2) | 993 | (11.8) | 2,983 | (75.0) | 993 | (25.0) |  | |
| NHI Self-employed | 22,792 | (88.8) | 2,880 | (11.2) | 8,645 | (75.0) | 2,880 | (25.0) | 0.00034 | |
| NHI Employee | 55,852 | (88.7) | 7,113 | (11.3) | 21,330 | (75.0) | 7,113 | (25.0) | -0.00057 | |
| **Region** |  |  |  |  |  |  |  |  |  | |
| Seoul | 14,439 | (89.4) | 1,711 | (10.6) | 5,126 | (75.0) | 1,711 | (25.0) |  | |
| Gyeonggi | 14,721 | (89.0) | 1,825 | (11.0) | 5,471 | (75.0) | 1,825 | (25.0) | -0.00032 | |
| Metropolitan | 22,405 | (88.9) | 2,809 | (11.1) | 8,429 | (75.0) | 2,809 | (25.0) | 0.00014 | |
| Rural | 34,525 | (88.2) | 4,641 | (11.8) | 13,932 | (75.0) | 4,641 | (25.0) | 0.00055 | |
| **CCI** |  |  |  |  |  |  |  |  |  | |
| 0 | 36,975 | (89.3) | 4,416 | (10.7) | 13,247 | (75.0) | 4,416 | (25.0) |  | |
| 1 | 18,977 | (88.9) | 2,378 | (11.1) | 7,133 | (75.0) | 2,378 | (25.0) | -0.00007 | |
| 2 | 12,835 | (88.3) | 1,701 | (11.7) | 5,101 | (75.0) | 1,701 | (25.0) | -0.00017 | |
| ≥3 | 17,303 | (87.4) | 2,491 | (12.6) | 7,477 | (75.0) | 2,491 | (25.0) | 0.00030 | |
| **Index year** |  |  |  |  |  |  |  |  |  | |
| 2008 | 10,768 | (90.1) | 1,187 | (9.9) | 3,566 | (75.0) | 1,187 | (25.0) | 0.00043 | |
| 2009 | 10,608 | (89.6) | 1,226 | (10.4) | 3,673 | (75.0) | 1,226 | (25.0) |  |  |
| 2010 | 8,637 | (89.5) | 1,016 | (10.5) | 3,050 | (75.0) | 1,016 | (25.0) |  |  |
| 2011 | 3,846 | (89.5) | 453 | (10.5) | 1,361 | (75.0) | 453 | (25.0) |  |  |
| 2012 | 2,523 | (89.0) | 311 | (11.0) | 935 | (75.0) | 311 | (25.0) |  |  |
| 2013 | 2,468 | (88.8) | 312 | (11.2) | 936 | (75.0) | 312 | (25.0) |  |  |
| 2014 | 2,409 | (88.7) | 306 | (11.3) | 921 | (75.1) | 306 | (24.9) |  |  |
| 2015 | 2,994 | (88.4) | 391 | (11.6) | 1,173 | (75.0) | 391 | (25.0) |  |  |
| 2016 | 10,560 | (88.1) | 1,425 | (11.9) | 4,274 | (75.0) | 1,425 | (25.0) |  |  |
| 2017 | 13,549 | (87.9) | 1,870 | (12.1) | 5,607 | (75.0) | 1,870 | (25.0) |  |  |
| 2018 | 17,728 | (87.7) | 2,489 | (12.3) | 7,462 | (75.0) | 2,489 | (25.0) |  |  |
| ** PSM: Propensity score matching; CCI: Charlson comorbidity index* | | | | | | | | | |  |
